# Differential basal expression of immune genes confers *Crassostrea gigas* resistance to Pacific oyster mortality syndrome

**DOI:** 10.1186/s12864-020-6471-x

**Published:** 2020-01-20

**Authors:** Julien de Lorgeril, Bruno Petton, Aude Lucasson, Valérie Perez, Pierre-Louis Stenger, Lionel Dégremont, Caroline Montagnani, Jean-Michel Escoubas, Philippe Haffner, Jean-François Allienne, Marc Leroy, Franck Lagarde, Jérémie Vidal-Dupiol, Yannick Gueguen, Guillaume Mitta

**Affiliations:** 1 IHPE, Université de Montpellier, CNRS, Ifremer, Université de Perpignan Via Domitia, Place E. Bataillon, CC080, 34095 Montpellier, France; 2 Ifremer, LEMAR UMR 6539, UBO/CNRS/IRD/Ifremer, 11 presqu’île du vivier, 29840 Argenton-en-Landunvez, France; 3 Ifremer, UMR 241 Écosystèmes Insulaires Océaniens, Labex Corail, Centre Ifremer du Pacifique, BP 49, 98725 Tahiti, French Polynesia; 4 Ifremer, Laboratoire de Génétique et Pathologie des Mollusques Marins, Avenue du Mus de Loup, 17930 La Tremblade, France; 5 MARBEC, Université de Montpellier, CNRS, IRD, Ifremer, 87 Avenue Jean Monnet, 34200 Sète, France

**Keywords:** Pacific oyster, Oyster disease, Resistance, OsHV-1, Antiviral molecular pathways, Invertebrate immunity

## Abstract

**Background:**

As a major threat to the oyster industry, Pacific Oyster Mortality Syndrome (POMS) is a polymicrobial disease affecting the main oyster species farmed across the world. POMS affects oyster juveniles and became panzootic this last decade, but POMS resistance in some oyster genotypes has emerged. While we know some genetic loci associated with resistance, the underlying mechanisms remained uncharacterized. So, we developed a comparative transcriptomic approach using basal gene expression profiles between different oyster biparental families with contrasted phenotypes when confronted to POMS (resistant or susceptible).

**Results:**

We showed that POMS resistant oysters show differential expression of genes involved in stress responses, protein modifications, maintenance of DNA integrity and repair, and immune and antiviral pathways. We found similarities and clear differences among different molecular pathways in the different resistant families. These results suggest that the resistance process is polygenic and partially varies according to the oyster genotype.

**Conclusions:**

We found differences in basal expression levels of genes related to TLR-NFκB, JAK-STAT and STING-RLR pathways. These differences could explain the best antiviral response, as well as the robustness of resistant oysters when confronted to POMS. As some of these genes represent valuable candidates for selective breeding, we propose future studies should further examine their function.

## Background

Originally from Asia, the Pacific oyster (*Crassostrea gigas*) has been introduced to numerous countries throughout the world (Canada, USA, Australia, New-Zealand, Chile, Mexico, Argentina, Brazil, South Africa, Namibia and in numerous European countries including France) during the twentieth century and has become the main oyster species farmed worldwide [1]. For decades, *C. gigas* has suffered from mortalities, but the severity of these outbreaks has dramatically increased since 2008. These outbreaks mainly affect juvenile stages, decimating up to 100% of young oysters in French farms [2]. In recent years, this mortality syndrome, designated Pacific oyster mortality syndrome (POMS), has became panzootic, being observed in all coastal regions of France and numerous other countries worldwide [3]. Today, POMS consequences are dramatic and represents a significant threat for the global oyster industry [2]. Research efforts have revealed a series of factors contributing to the disease, including infectious agents interacting with seawater temperature and oyster genetics [2, 4–8]. Recently, holistic molecular approaches performed on susceptible and resistant families of oysters, deciphered the mechanism of POMS by combining dual RNAseq (oyster, OsHV-1 and vibrio), 16S rDNA metabarcoding, histology and invalidation of virulence genes from bacteria [9, 10]. These studies showed that an infection by the Ostreid herpesvirus (Ostreid herpesvirus type 1 μVar) is the critical step in the infectious process leading to an immune-compromised state by altering hemocyte physiology [9]. This first process is followed by a microbiota destabilization which “opens the door” to bacterial pathogens (e.g. vibrios) that target hemocyte to induce their lysis [10]. The infectious process is completed with subsequent bacteraemia, which is the ultimate step inducing oyster death [9].

However, some oysters are disease-resistant to POMS. Genetic studies on oyster resistance revealed a significant additive genetic component for survival during OsHV-1 infection [4, 11, 12]. Over the past decade, many genomic resources have been developed including a reference genome [13] and SNP arrays [14]. These resources enabled an investigation of the genetic architecture of *C. gigas* resistance to OsHV-1 infection; juvenile oysters were experimentally challenged with OsHV-1 and genotyped using a high density linkage map constructed for the Pacific oyster [15]. The genome-wide association developed suggested a polygenic nature of resistance to OsHV-1 and highlighted region of linkage group 6 containing a significant QTL affecting host resistance [15]. Several SNPs showing an association with survival and/or viral load were located in several genes encoding a RAN Binding Protein 9, a Coronin and an actin motor protein Myo10. However, their involvement in the resistance process remains unknown. A recent transcriptomic approach employed on different oyster biparental families displaying contrasted susceptibilities to POMS showed that the early induction of genes involved in antiviral defense is a hallmark of resistant families [9]. However, the genetic components responsible for this early induction remain unidentified.

To fill this gap in knowledge and to identify putative transcriptomic determinants associated to POMS resistance, we compared the basal transcriptomes of six biparental families of oysters displaying contrasted susceptibilities to the disease (3 resistant and 3 susceptible). Here, we showed that genes involved in stress response, protein modifications, maintenance of DNA integrity and repair, and immune and antiviral pathways are differentially expressed in resistant oysters.

## Results

### Infectious environments select oysters resistant to POMS

**Fig. 1:**
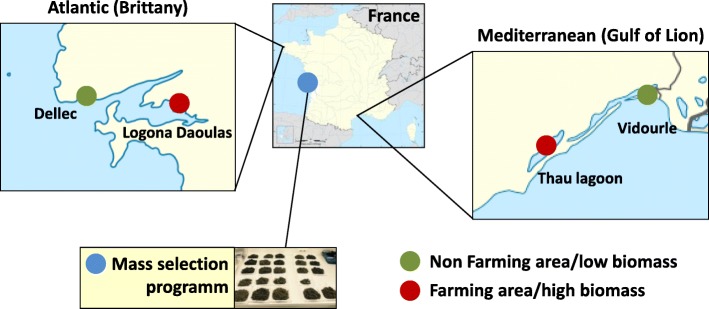
Broodstock origins for the production of biparental oyster families. Wild stocks were sampled in farming (purple) and non-farming (orange) sites in two geographic areas (Atlantic and Mediterranean coasts). Mass selected oysters (yellow) originated from the Ifremer hatchery of La Tremblade [16]. Image source: commons.wikimedia.org

**Fig. 2:**
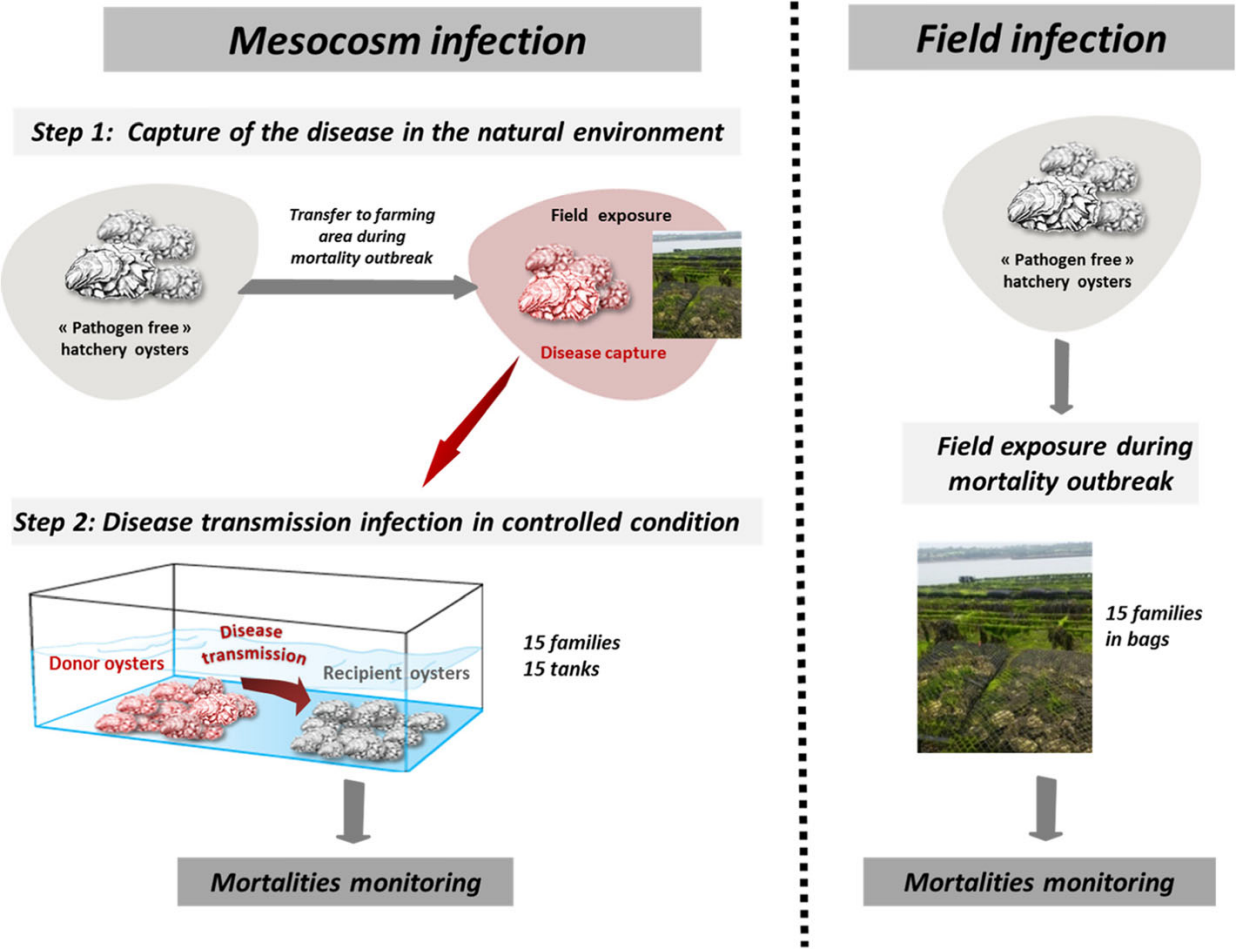
Schematic of the mesocosm (left panel) and field (right panel) protocols of infection. For the experimental infection, pathogen free oysters (mix of the fifteen families) were deployed in the natural environment in a farming area during disease outbreaks and brought back to a controlled environment to transfer the disease to each of the 15 oyster families under controlled conditions. For field infection, each of the 15 oyster families were exposed to an infectious environment during a disease outbreak. Experimental infections were performed with infectious environments from Atlantic and Mediterranean origin

In 2015, oyster broodstocks were collected in two distinct geographic areas (Brittany-Atlantic coast and Gulf of lion- Mediterranean coast) and two sampling sites (farming, high biomass and non-farming, low biomass) from each area (Fig. 1). These broodstocks were used to produce 12 biparental families (3 families from each origin and sampling sites). Three additional biparental families were produced from broodstocks originating from a mass selection program for higher survival for POMS [16] (Fig. 1). These 15 oyster families were subjected to four infectious challenges performed with two infectious environments (Atlantic and Mediterranean) and two experimental procedures (mesocosm and field infections) (Fig. 2 and Table 1).

**Table 1:** Oysters broodstock origin and families susceptibility to the mesocosm and field infection trials

Oyster Family	Broodstock origin			Mortality (%)			
	Geographic area	Sampling site	Geographical coordinate	Atlantic mesoscosm infection	Atlantic field infection	Mediterranean mesocosm infection	Mediterranean field infection
F1	Atlantic	Farming area	Logonna Daoulas (lat 48.335263 - long - 4.317922)	98	99	75	89
F2	Atlantic	Farming area	Logonna Daoulas (lat 48.335263 - long - 4.317922)	68	100	70	88
F9	Atlantic	Farming area	Logonna Daoulas (lat 48.335263 - long - 4.317922)	56	95	14	44
F11	Atlantic	Non farming area	Dellec (lat 48.353970, long - 4.566123)	99	100	90	93
F14	Atlantic	Non farming area	Dellec (lat 48.353970, long - 4.566123)	96	100	94	94
F15	Atlantic	Non farming area	Dellec (lat 48.353970, long - 4.566123)	100	100	97	98
F21	Breeding program	Breeding program	Charente Maritime- La Tremblade (lat 45.781741, long - 1.121910)	3	2	1	7
F23	Breeding program	Breeding program	Charente Maritime- La Tremblade (lat 45.781741, long - 1.121910)	12	24	12	11
F28	Breeding program	Breeding program	Charente Maritime- La Tremblade (lat 45.781741, long - 1.121910)	32	38	18	28
F32	Mediterranean	Non farming area	Vidourle (lat 43.553906, long 4.095175)	56	98	78	89
F33	Mediterranean	Non farming area	Vidourle (lat 43.553906, long 4.095175)	48	92	39	59
F37	Mediterranean	Non farming area	Vidourle (lat 43.553906, long 4.095175)	30	95	2	30
F42	Mediterranean	Farming area	Thau lagoon (lat 43.418736, long 3.622620)	37	96	44	65
F44	Mediterranean	Farming area	Thau lagoon (lat 43.418736, long 3.622620)	40	86	2	10
F48	Mediterranean	Farming area	Thau lagoon (lat 43.418736, long 3.622620)	18	40	7	12

High variability in percentages of mortality, ranging from 1 to 100%, was observed among families (Table 1). Family F15 showed the most susceptibility with a mortality rate higher than 97% for any infection trial. In contrast, Family F21 showed the highest resistance whatever the infection trial. Taking into account the 15 families and the 2 infectious environments, the percentage of mortalities observed in the field were not significantly different than those obtained in mesocosm conditions (Mann-Whitney test, *p* = 0.06). Overall, disease susceptibility was quite similar for the different families in the different infection trials (Table 1). Mortalities observed for the 15 families were 1.75 fold more important for the Atlantic infectious environment than for the Mediterranean infectious one (Mann Whitney, *p* = 0.02) (Table 1).

**Fig. 3:**
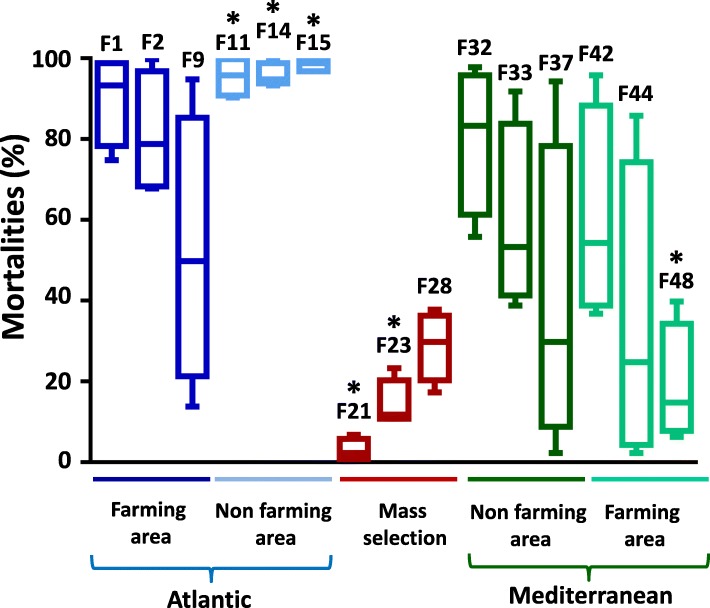
Mean mortality of each oyster family in different infection trials. The broodstocks origin – blue: Atlantic, green: Mediterranean, dark: Farming/high biomass, light: Non farming/low biomass, and red: mass selection program - are indicated below the graph. Each box plot represents the mean mortality and the variance for each biparental family submitted to the four infection trials. The three most susceptible and the three most resistant oyster families used for transcriptome analyses are indicated by asterisks

**Fig. 4:**
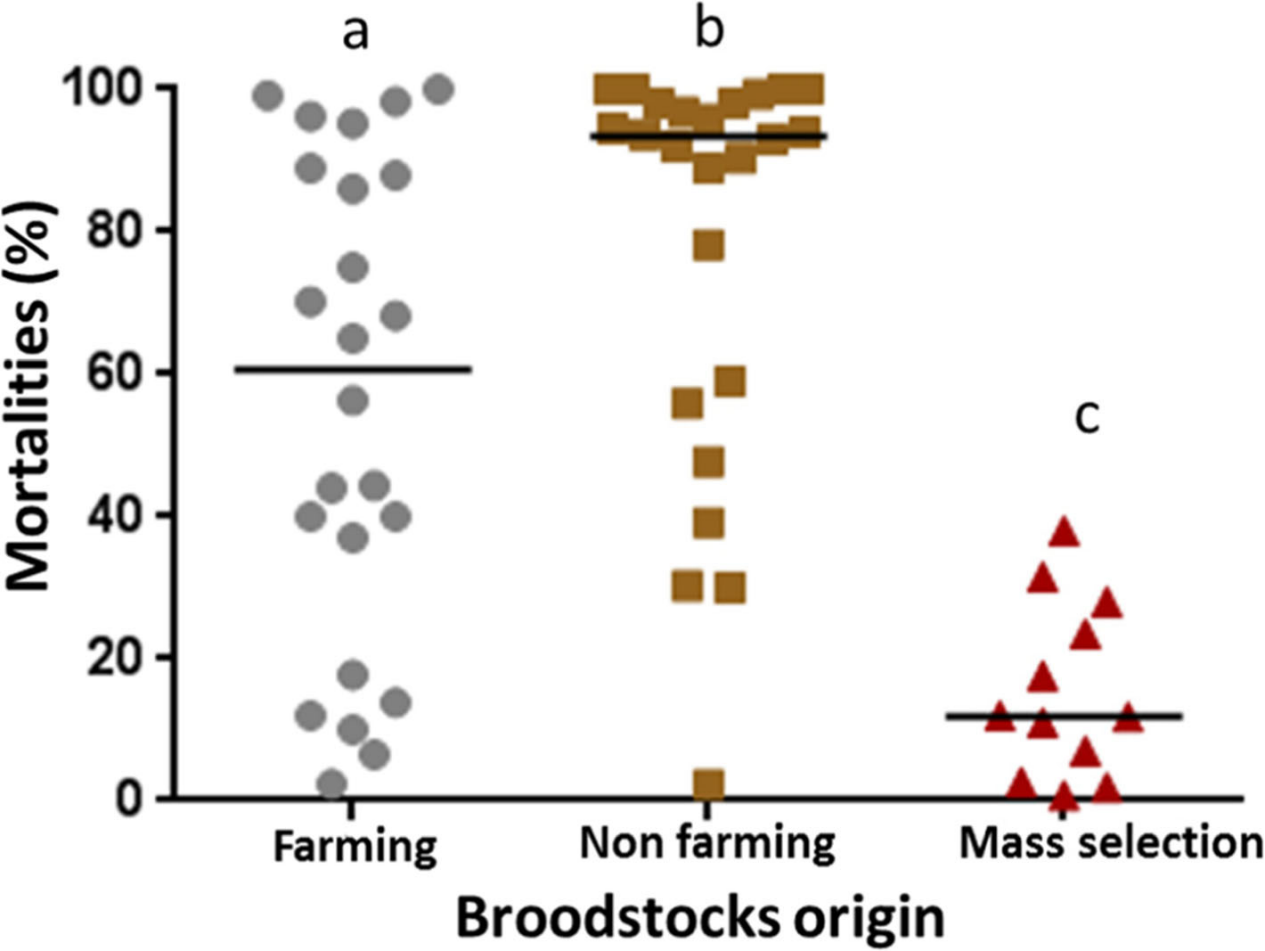
Oyster families produced from broodstocks surviving POMS challenge are more disease resistant. Mortalities of the different families after exposure to POMS in the different infection trials were analysed by origin of the broodstocks (farming/high biomass, and non-farming/low biomass area or mass selection program). Significant differences between conditions are indicated by different lowercase letters (different letters indicate significant difference, a, b or c; Mann-Whitney U test, *p* < 0.05)

As expected, the 3 families (F21, F23 and F28) produced from broodstocks coming from the mass selection program [16] displayed low percentages of mortality (Figs. 3 and 4). Similarly, families obtained from wild oysters sampled in a farming area and putatively submitted to disease selection were also more resistant than those from non-farming areas ((Mann Whitney, *p* = 0.018), Fig. 4).

To confirm POMS disease in the different mesocosm experiments, we quantified OsHV-1 and total vibrio loads by qPCR (Additional file 1). We observed the colonization of oyster flesh by OsHV-1 and vibrios in both Atlantic and Mediterranean mesocosm experiments 72 h post-exposure.

The 6 families, retained for the subsequent comparative transcriptomics, were the three best oyster families for POMS resistance (F21, F23 and F48 renamed R_F21_, R_F23_ and R_F48_, respectively) and the three worst (F11, F14 and F15, renamed S_F11_, S_F14_ and S_F15_, respectively) (Fig. 3).

### Stress and immune functions are enriched in the basal transcriptome of resistant oysters

To identify putative transcriptomic determinants associated with POMS resistance, we compared the basal transcriptome profiles of the 6 selected families (R_F21_, R_F23_, R_F48,_ S_F11_, S_F14_ and S_F15_), maintained in the same hatchery conditions and without disease challenge. We sequenced a total of 36 RNA-seq libraries (6 families, 2 independent experiments and 3 replicates for each experiment). Sequencing yielded between 30.1 and 39.3 million Illumina single reads per sample of which 70.1–74.9% mapped to the *C. gigas* V9 reference genome.

**Fig. 5:**
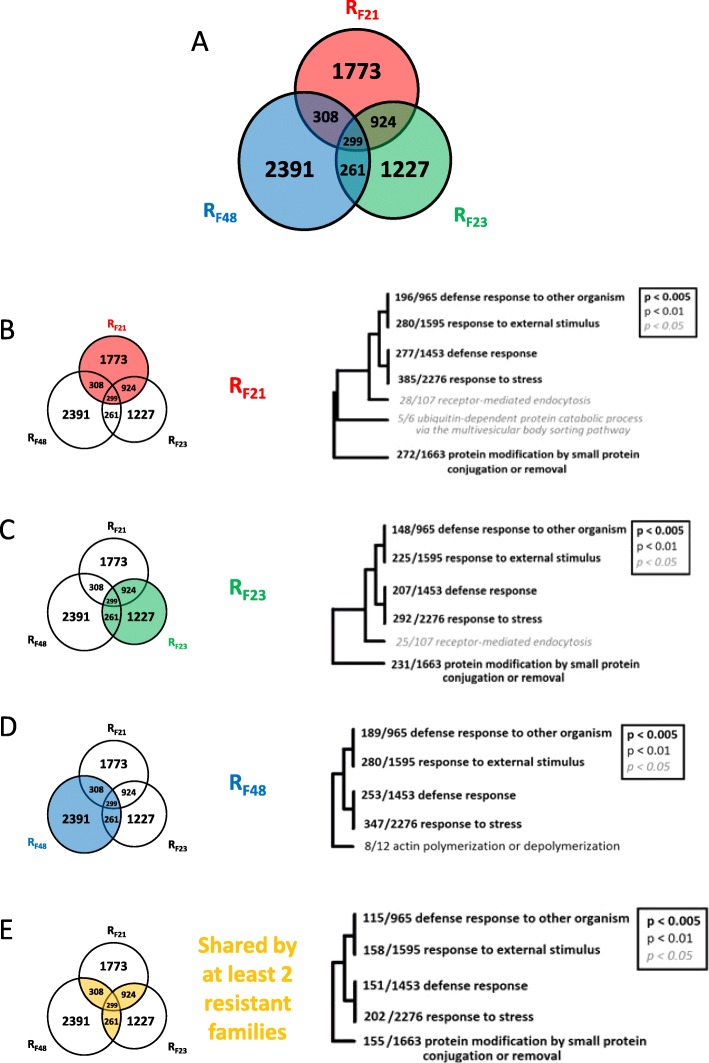
Venn diagram of DEGs between resistant and susceptible oyster families and enrichment analysis. **a** Venn diagram of DEGs between resistant and susceptible oyster families, where each circle corresponds to a resistant family (R_F21_ in red, R_F23_ in green or R_F48_ in blue). The numbers inside indicate the number of DEGs between each resistant family and the three susceptible families (S_F11_, S_F14_ and S_F15_). The numbers in overlapped circles indicate the numbers of DEGs commons to two or three resistant families. A total of 7183 DEGs were identified. Hierarchical clustering trees of GO categories (biological process) significantly enriched for the (**b**) 3304 DEGs of the R_F21_ family, (**c**) 2711 DEGs of the R_F23_ family, (**d**) 3259 DEGs of the R_F48_ family and (**e**) 1792 DEGs shared by at least two resistant families. The ratio before each GO category represents the number of DEGs in this category divided by the total number of genes related to this category annotated in the genome

**Fig. 6:**
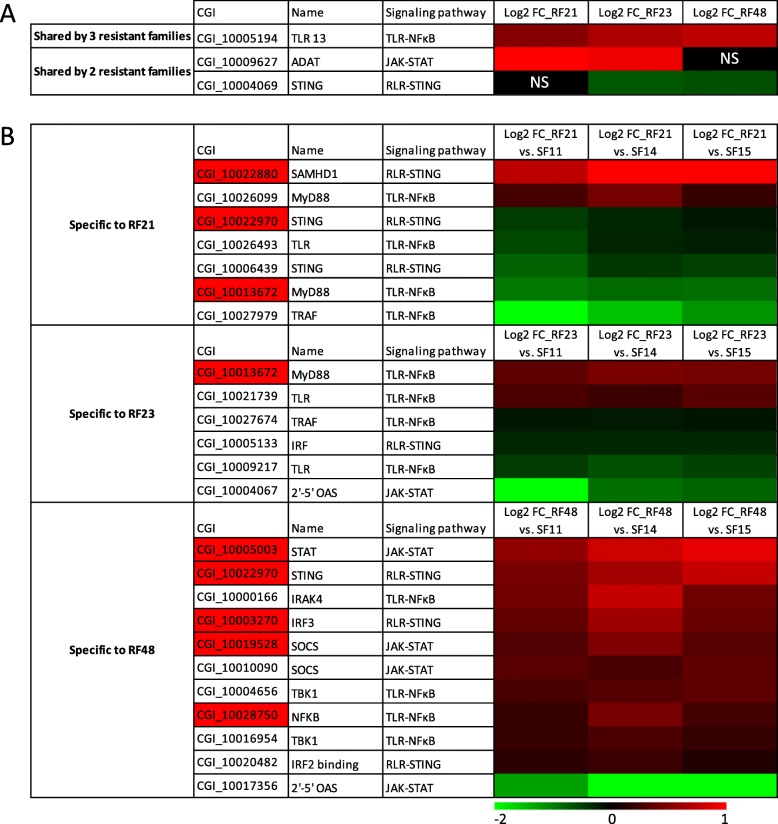
DEGs related to TLR-NFκB, JAK-STAT and RLR-STING pathways. **a** DEGs shared by at least two resistant oyster families. Each resistant family (R_F21_, R_F23_ and R_F48_) is compared to the three susceptible families (S_F11_, S_F14_ and S_F15_); mean log2 fold change is given. **b** DEGs specific to each resistant family. Each resistant family (R_F21_, R_F23_ and R_F48_) is compared to the three susceptible families separately (S_F11_, S_F14_ and S_F15_). The intensity of the colour from green to red indicates the magnitude of the log2 fold change for the corresponding transcript. NS: not significant. CGI indicated in red are also associated to an early antiviral response of resistant oysters [9]

From these RNA-seq data, we compared the basal transcriptome of each resistant family to the three susceptible families using DEseq (DEseq *p-value* < 0.05). The differentially expressed genes (DEGs) common between each comparison were retained for further analysis. This strategy identified 3304, 2711 and 3259 DEGs modulated in the same way (up- or down-represented for the three comparisons) in R_F21_, R_F23_ and R_F48_, respectively (Fig. 5a). Among these DEGs, (i) 299 were differentially expressed by the three resistant families, (ii) 924 were differentially expressed by both R_F21_ and R_F23_, (iii) 261 were differentially expressed by both R_F23_ and R_F48_, and (iv) 308 were differentially expressed by both R_F21_ and R_F48_ (Fig. 5a and Additional file 2). The remaining 1773, 1227 and 2391 DEGs displayed a specific differential expression in R_F21_, R_F23_ and R_F48_, respectively (Fig. 5a and Additional file 3, Additional file 4 and Additional file 5). A previous study has evidenced that the resistance to POMS is associated to an early antiviral response that blocks OsHV-1 replication [17–19]. Indeed, 308 DEGs associated to antiviral defence are early induced in resistant oysters and among them, 61 are differentially expressed at basal level between resistant and susceptible families in the present study. These genes are highlighted in red in the Additional file 2, Additional file 3, Additional file 4, and Additional file 5. A part of them are related to TLR-NF-κB, JAK-STAT and RLR-STING antiviral signalling pathways (indicated in red in Fig. 6). To determine the enriched functions, we used a gene ontology (GO) enrichment analysis. As the mechanisms underlying resistance can be specific or shared by oyster families, we performed the enrichment analysis on DEGs for each resistant family separately and also on DEGs shared by at least two of the three resistant families. First, the analyses performed on DEGs of each resistant family separately showed a limited number of enriched functions (7, 6 and 5 for R_F21_, R_F23_ and R_F48_, respectively; Fig. 5b, c and d, respectively). Interestingly, four functional categories (“defense response to other organism”, “response to external stimulus”, “defense response” and “response to stress”) were enriched for the 3 resistant families. It is noteworthy that R_F21_ and R_F23_ shared two additional enriched categories (“receptor-mediated endocytosis” and “protein modification by small protein conjugation or removal”). Finally, a functional category related to the “actin polymerization and depolarization” showed enrichment in R_F48_ only, while a functional category related to “ubiquitin-dependent protein catabolic process” showed enrichment in R_F21_ only. Second, the GO enrichment analysis on DEGs shared by at least two of the three resistant families (1792 DEGs, Additional file 2) revealed five enriched functional categories (Fig. 5e): “defense response to other organism”, “response to external stimulus”, “defense response”, “response to stress” and “protein modification by small protein conjugation or removal”. DEGs falling in these five enriched categories (374 genes) are shown in Additional file 2 (CGI indicated in yellow).

### Resistant oysters differentially express common and specific immune genes

To further delineate the molecular mechanisms underlying POMS resistance shared by the different resistant families, we first analysed the 374 DEGs belonging to the above identified functions (*ie.* defense response, response to stress, defense response to other organism, response to external stimulus and protein modification by small protein conjugation or removal) and shared by at least two of the three resistant families (CGI indicated in yellow in Additional file 2). Among these genes, we found members of large multigene families known to be involved (i) in stress response like heat shock proteins (HSP) and glutathione S-transferases, (ii) in protein modifications like ubiquitin ligases and Tripartite Motif containing proteins (TRIM), (iii) in maintenance of DNA integrity and repair like Poly [ADP-ribose] polymerases (PARP), nucleases and helicases, (iv) in PAMP (Microbe Associated Molecular Pattern) recognition (PRR) like C1q domain containing proteins, lectins, scavenger receptors (SR), Fibrinogen domain containing proteins, hemagglutinins and (v) in antiviral defense like IFI44 proteins. We also identified a series of genes putatively involved in antiviral defense and signaling (TLR-NF-κB, JAK-STAT and RLR-STING pathways, Fig. 6a). A putative endosomal Toll-like receptor 13 was overexpressed in the 3 resistant families. A tRNA adenosine deaminases (ADAT) was over-represented in R_F21_ and R_F23_. A stimulator of interferon genes (STING) is under-represented in R_F23_ and R_F48_ (see Fig. 6a for CGI number for each DEG).

Finally, we analyzed DEGs for each resistant family belonging to the enriched categories described in Fig. 5 (i.e. defense response, response to stress, defense response to other organism, response to external stimulus, protein modification by small protein conjugation or removal, receptor-mediated endocytosis, ubiquitin-dependent protein catabolic process via the multivesicular body sorting pathway and actin polymerization or depolymerisation; the corresponding CGIs are highlighted in yellow in Additional file 3, Additional file 4, and Additional file 5). This analysis highlighted specific processes associated with resistance in each resistant genotype. These genes represented 371, 251 and 315 DEGs in R_F21_, R_F23_ and R_F48_, respectively. In these specific sets of DEGs, we again found several genes belonging to the same large multigene families reported above (HSP, glutathione S-transferases, ubiquitin ligases, TRIM, PARP, nucleases, helicases, PRR and IFI44). In addition, several genes involved in antiviral and signaling pathways were also found differentially expressed in each resistant family specifically (TLR-NF-κB, JAK-STAT and RLR-STING pathways, Fig. 6b). Transcripts corresponding to a Toll-like receptor (TLR), 2 myeloid differentiation primary response 88 (MyD88), a TNF receptor-associated factor (TRAF), a deoxynucleoside triphosphate triphosphohydrolase (SAMHD1), and 2 stimulator of interferon genes (STING) were differentially represented in the R_F21_ family (see Fig. 6b for CGI number for each DEG). Transcripts corresponding to 2 TLRs, a MyD88, a TRAF, a 2′,5′- oligoadenylate synthase (2′,5′-OAS) and an interferon regulatory factor (IRF), were differentially represented in the R_F23_ family (see Fig. 6b for CGI number for each DEG). However, in these 2 families, the majority (9/13) of these DEGs was under-represented in comparison with susceptible oysters (Fig. 6b). In contrast, the majority (10/11) of DEGs in the R_F48_ family was over-represented in this resistant family. They corresponded to an interleukin-1 receptor-associated kinase (IRAK), a NF-κB p105 subunit, 2 Serine threonine- kinases TBK1, a signal transducer and transcription activator (STAT), 2 suppressors of cytokine signaling (SOCS), a STING and 2 IRFs (see Fig. 6b for CGI number for each DEG). Only a 2′,5′- oligoadenylate synthase (OAS) appeared under-represented in R_F48_.

## Discussion

Fifteen oyster families were produced and phenotyped using mesocosm and field infections. No significant differences in terms of mortality between mesocosm and field infections were evidenced. This result suggested that the disease developed in mesocosm experiments accurately reproduces the disease in the natural environment with the same outcomes. The mortalities observed with the Atlantic infectious environment were significantly higher than those obtained from Mediterranean one, while OsHV-1 and total vibrio colonised oyster tissues in both experiments. Differences in the two infectious environments could explain these differences in mortalities. Indeed, the presence of different OsHV-1 virus variants, already reported in different infectious environments [20], could explain the differences observed. Future studies will investigate this specific question. A second explanation can be the age difference of oysters submitted to these two infectious environments. Indeed, the oysters submitted to the Mediterranean infections were 2 months older. As resistance to the disease increases with age [4, 21], we cannot exclude that older oysters were slightly less susceptible. As expected, higher survival rates occurred in oyster families produced from broodstocks coming from a breeding program using mass selection [16] or from broodstocks recruited in farming areas compared to those from broodstocks recruited in non-farming areas. This last result shows that the selective pressure exerted by the infectious environment during the first years after recruitment is sufficient to select oysters more resistant to POMS. Indeed, selection for OsHV-1 has a genetic basis [22, 23]. In contrast, wild oysters collected in non-farming areas still showed high susceptibility to POMS suggesting either the absence of disease pressure or a much lower disease pressure than in farming area as previously shown [24].

In order to identify the transcriptomic determinants of POMS resistance, we selected the three most resistant (R) and susceptible (S) oyster families and compared their basal transcriptome when they were maintained in the same controlled conditions and without exposure to the disease. Two R families came from broodstocks selected through “mass selection” (R_F21_ and R_F23_), and one R family came from broodstocks recruited in a farming area and selected through “natural selection” (R_F48_). The three S families (S_F11_, S_F14_ and S_F15_) were produced from broodstocks recruited in non-farming areas. Our transcriptomic analysis revealed a clear modulation and enrichment of genes belonging to functions related to defense, stress and protein modifications among the resistant and susceptible families. A series of genes related to these functional categories belong to large multigene families like Tripartite Motif containing proteins (TRIM), ubiquitin ligases, IFI44, heat shock proteins, glutathione S-transferases, proteins involved in maintenance of DNA integrity and repair like Poly [ADP-ribose] polymerases (PARP), nucleases and helicases, C1q domain containing proteins, lectins, scavenger receptors, fibrinogen domain containing proteins and hemagglutinins. Other genes involved in immune and antiviral pathways (TLR-NF-κB, JAK-STAT and RLR-STING) were also found to be modulated in our study. Such differences in basal expression of immune genes have been already described in resistant and susceptible cultivars of litchi, apple or soybean to pathogens [17–19]. Variations of immune status between human populations have also been described at genetic and epigenetic levels, and these changes modulate several key regulators of innate immunity [25, 26]. These differences in immune status likely arise from the different selected pressures experienced that impact the host response to pathogens, especially in African populations, which develop a strong inflammatory response compared to European populations [25]. Even if the genetic determinism of POMS resistance is clear [4, 11, 12], we cannot exclude that the difference in expression reported in our study may be controlled by epigenetic mechanisms known to influence gene expression in invertebrates [27]. This possibility shall be investigated in future studies.

As R_F21_, R_F23_ and R_F48_ can control virus replication in oyster tissues [9], we focussed particularly in this discussion on genes belonging to pathways clearly involved in oyster antiviral defense [28]. Several of the multigene families identified are potentially implicated in antiviral immunity. TRIM, one of the most represented multigene family in our sets of DEGs (one third), can target viral proteins for ubiquitination, in association with ubiquitin ligases (also identified here), to inhibit viral replication and induce RLR-STING and TLR-NF-κB signaling pathways, which contribute to antiviral defense [29]. IFI44 multigene family are interferon-alfa inducible proteins, which are associated with infection of several viruses and can affect viral replication [30]. Some other genes like PARP could also participate to the defense against viral infection [31]. As TLR-NF-κB, JAK-STAT and RLR-STING pathways are 3 conserved pathways crucial to mount an efficient antiviral response [28], we made a particular focus on the DEGs belonging to these pathways. Only transcripts corresponding to one gene belonging to these pathways is overrepresented in the 3 resistant families. It corresponded to an endosomal Toll-like Receptor displaying similarities to the TLR 13, which can act as a sensor of viral and bacterial RNA in the TLR-NF-κB signalling pathway [32, 33]. This gene is particularly interesting because its sensor function could explain how these three resistant family may detect the viral infection earlier to mount a more rapid and efficient antiviral response, which is a common feature of these three resistant family [9]. Another gene, a tRNA adenosine deaminases (ADAT), displayed an over-representation of its transcripts in R_F21_ and R_F23_. ADAT (tRNA adenosine deaminases) gene is the ancestral form of ADAR (dsRNA-specific adenosine deaminase). ADAR has been recently described in *C. gigas* to be highly induced after OsHV-1 infection and potentially mediate editing (A to I) impacting RNAs expressed by OsHV-1 [34]. ADAT has also an I to A editase domain which could potentially edit OsHV-1 RNAs [34]. However, the anti- or pro-viral activity of this editing remains unknown in the case of OsHV-1 infection [34]. Considering their putative antiviral role, TLR 13 and ADAT represent good candidates whose function should be examined in future studies. In addition, a series of genes corresponding to the TLR-NF-κB, JAK-STAT and RLR-STING pathways were differentially expressed only in one family. Overall, we obtained very different results for this set of DEGs between families produced from “mass selection” (R_F21_ and R_F23_) and “natural selection” (R_F48_) broodstocks. Indeed, most transcripts corresponding to these pathways were over-represented in the R_F48_ family. They correspond to IRAK, NF-kB and TBK1 (TLR-NF-κB pathway), STAT and SOCS (JAK-STAT pathway) and IRFs and STING (RLR-STING pathway) genes. For the two selected families produced from “mass selection” (R_F21_ and R_F23_), genes belonging to these molecular pathways were also identified. The majority of the corresponding transcripts were under-represented (2 TLRs, a MyD88, 2 TRAF, 2 STING, a 2′,5′-OAS and an IRF), while a minor part of them were over-represented (a TLR, 2 MyD88, and a SAMHD1). Thus, the molecular phenotype of the R_F21_ and R_F23_ families differs in part from that of the family R_F48_. R_F21_ and R_F23_ families are derived from a four-generation selection program conducted in natural environment showing a significant positive response to selection with a gain of resistance/survival that accumulated over the generations [16]. Consequently, the R_F21_ and R_F23_ families might develop mechanisms of resistance and a genetic architecture for this trait that are significantly different by comparison with the R_F48_ family whose genitors were confronted to a single POMS outbreak. We propose this rationale could explain the differences in the transcriptomic phenotype observed.

Interestingly, several DEGs commons and specifics to resistant oyster families have been previously associated to an early antiviral response of resistant oysters [15]. Among these DEGs, several genes related to antiviral signalling pathways (TLR-NF-κB, JAK-STAT and RLR-STING pathways) are evidenced. Thus, both an over-representation at basal level and an up-regulation in early phase of infection of genes related to antiviral signalling pathways could confer resistance to POMS.

Overall, our results show that the selection process in these different oyster families has impacted their molecular phenotype in numerous molecular pathways, particularly for genes involved in functions related to antiviral immunity and maintenance of DNA integrity and repair. These modifications could participate in improving their fitness when confronted to a viral infection. Several identified DEGs were modulated in at least 2 disease resistant families, but most of them were differentially expressed in only a single family. Taken together, these results suggest that resistance mechanisms can vary at least partially among genotypes and that they are probably complex (multigenic). This is in agreement with a recent study of Gutierrez and collaborators [15], which suggested a polygenic nature of oyster resistance to OsHV-1.

## Conclusions

A previous study demonstrated that POMS resistant oyster families present a more rapid antiviral response compared to susceptible oyster families [9]. This rapid antiviral response of resistant oysters blocks replication of the herpes virus OsHV-1 and prevents subsequent bacteraemia by opportunistic bacterial pathogens [9]. Here, we found differences in basal expression levels of genes related to immunity suggesting different immune status between resistant and susceptible oysters. These expression differences occurred for genes that mediate stress responses, protein modifications, maintenance of DNA integrity and repair, and immune and antiviral pathways, including sensors. These differences could explain the early antiviral response, as well as the robustness of resistant oysters when confronted to POMS. Further studies will determine the function of these promising candidates and uncover the link between these expression differences and disease resistance. Such functional studies must precede identifying valuable candidates for future successful selective breeding.

## Methods

### Production of biparental oyster families

We collected wild stocks of *Crassostrea gigas* in farming as well as non-farming areas in two regions (French Mediterranean and Atlantic coasts) (Fig. 1). In addition, a fifth stock used selected oysters for their higher resistance to the infection by OsHV-1 [16]. From each stock, three bi-parental families were produced as previously described [9]. The 15 oyster families were maintained under controlled biosecured conditions at the Ifremer laboratory of Argenton (Brittany, France; lat 48.521536, long − 4.767799) to ascertain that no oyster pathogens would interfere with further experiments. The “pathogen-free” status of the animals was confirmed by i) the absence of OsHV-1 DNA detection by qPCR and ii) a low *Vibrio* presence (< 10 cfu/g tissue) determined by isolation on selective culture medium (thiosulfate-citrate-bile salts-sucrose agar, TCBS) [7]. Oysters were observed to remain free of any abnormal mortality throughout the larvae until the beginning of the experimental and field infections. No mortality was observed during the two last months preceding the beginning of the experiments.

### Mesocosm infections

The mesocosm infection protocol consists of cohabitation in controlled conditions between *C. gigas* oysters carrying the POMS disease (“donors”) and “pathogen-free” *C. gigas* oysters (“recipients”) [21]. A first experimental infection used donors previously exposed to the infectious environment of Atlantic origin. The donors were “pathogen-free” oysters (mixture of 116-day-old oysters from the 15 families, 17,700 g with a mean individual weight of 1.1 g) were first deployed in a farming area (Logonna Daoulas, lat 48.335263, long − 4.317922) during the infectious period until the first mortalities occurred (0.01%). This low percentage of mortality was sufficient to be certain that the oysters were diseased [7–9]. Then, donor oysters were transferred back to the laboratory and placed in contact with “pathogen-free” recipient oysters in a controlled environment (Fig. 2). The experiment was conducted using the same biomass (1120 g) of donors in cohabitation in 15 independent tanks (500 l), each containing one of the 15 families (recipient oysters with a mean individual weight of 1.1 g) which were previously acclimatized in these structures for two weeks. The Atlantic experimental infection began on 17 July 2015 and ended on 31 July 2015. Similarly, a second experimental infection used donors previously exposed to the infectious environment of Mediterranean origin in a farming area (Thau lagoon, lat 43.418736, long 3.622620), except that donors deployed were a mixture of 176-day-old oysters from the 15 families (26,500 g with a mean individual weight of 1.7 g) and that the biomass of donors and the biomass of recipients in each tank was 1760 g each (recipient oysters with a mean individual weight of 1.73 g). The Mediterranean experimental infection began on 21 September 2015 and ended on 6 October 2015.

### Field infections

Concomitantly to the mesocosm infections, the survival rates of the 15 oysters families (*n* = 100 per family) were also recorded in oyster farms in the two infectious environments where were deployed the donors (Fig. 2, Logonna Daoulas in Atlantic area and Thau lagoon in Mediterranean area). The Atlantic field infection began on July 17th 2015 and ended on August 3rd 2015 while it was on September 21st 2015 and October 6th 2015 for the Mediterranean.

### Statistical analyses

For Mesocosm and field infections, statistical data analysis was conducted in GraphPad Prism (V6.0) for Windows (GraphPad Software, La Jolla, USA). For all analysis, statistical significance was set at *p* < 0.05. We performed non-parametric Kruskal-Wallis tests to compare mortalities. When Kruskal-Wallis tests were significant, we computed pairwise comparisons using Mann-Whitney U t-test.

### Oyster transcriptome analyses

Before experimental infection, 10 oysters in triplicate were randomly sampled from each family without blinding protocols. The shell was removed, and pools of 10 oysters were flash frozen in liquid nitrogen. Oyster pools (10 individuals per pool) were ground in liquid nitrogen in 50-ml stainless steel bowls with 20-mm-diameter grinding balls (Retsch MM400 mill). The obtained powders (stored at − 80 °C) were then used for extracting RNA. RNA was extracted from powdered oysters using the Direct-Zol RNA Miniprep kit (Proteigene) according to the manufacturer’s protocol. RNA concentration and purity were checked using a Qubit® Fluorometer (Thermo Scientific), and their integrity was analysed by capillary electrophoresis on a BioAnalyzer 2100 (Agilent). RNA-seq library construction and sequencing were performed by the Bioenvironment platform (Perpignan, France). PolyA+ library preparation was performed from 500 ng total RNA using NEBNext Ultra II Directional RNA Prep Kit for Illumina (New England Biolabs) according to manufacturer’s instruction and sequenced on a NextSeq550 Instrument (SE 75 bp). All data treatments were carried out under a local galaxy instance [35]. Reads quality was checked using the Fastq-X toolkit [36] and since all reads display a Phred score above 26 over 90% of the their length no subsequent quality filtering was done. Adpator trimming was then performed using CutAdapt [37]. Paired-end mapping to the *C. gigas* reference genome (assembly version V9 [13]) was performed using RNAstar using default parameters (Galaxy Version 2.4.0d-2 [38]). The HTSeq-count was used to count the number of reads overlapping annotated genes. The parameters used were; mode = Union, Stranded = No, Minimum alignement quality = 10, Feature type = exon; ID attribute = gene_id; all other parameters used the default value (Galaxy Version v0.6.1) [39]. Finally, the differential gene expression levels were analysed with the DESeq2 R package [40]. Fold changes between each resistant and susceptible oysters were considered significant when the adjusted *p*-value (Padj) for multiple testing with the Benjamini-Hochberg procedure, which controls the false discovery rate (FDR), was *<* 0.05.

Thus, each resistant family (6 replicates) was compared to each susceptible family (6 replicates) separately (RF21 vs. S_F11_, R_F21_ vs. S_F14_, R_F21_ vs. S_F15_; R_F23_ vs. S_F11_, R_F23_ vs. S_F14_, R_F23_ vs. S_F15_; R_F48_ vs. S_F11_, R_F48_ vs. S_F14_, R_F48_ vs. S_F15_). For each resistant family, only DEGs significant in the 3 comparisons and in the same way (up- or down-represented for the three comparisons) were retained.

### Gene ontology annotation and enrichment analysis

To work with current functional annotations of the *C. gigas* gene set, we performed a functional annotation (Additional file 6). Blastx comparison against the NR database was performed for the 28,027 genes annotated in the genome, with a maximum number of target hits of 20 and a minimum e-value of 0.001. From these 20 hits, a percentage of mean similarity was calculated and we retained only results with mean similarity > 40%. XML blast result files were loaded onto Blast2GO [41] for GO mapping and annotation with the b2g_sep13 version of the B2G database. These results were used as inputs for GO enrichment analysis, which was performed using adaptive clustering and a rank-based statistical test (Mann-Whitney U-test combined with adaptive clustering). The R and Perl scripts used [42] can be downloaded [https://github.com/z0on/GO_MWU]. The following parameters were used for adaptive clustering: largest = 0.5; smallest = 5; clusterCutHeight = 0.25. For the continuous value characterization of each gene in the dataset, we used a strategy aiming to take into account both the level of expression and the significance of the differential expression. To combine these two factors, the log2 fold change was attributed to genes that were significantly differentially expressed (adjusted *p* < 0.05), while a zero was attributed to the others. A category was considered enriched with a FDR < 1%.

### DNA extraction and quantification of OsHV-1 and total *Vibrio*

DNA extractions were performed from the same samples used for RNA extraction using the Nucleospin tissue kit (Macherey-Nagel) according to the manufacturer’s protocol. In order to improve DNA extractions, we added a crushing step, which consisted in an additional 12 min mechanical lysis using zirconium beads (0.1 mm dia., BioSpec) before the 90 min enzymatic lysis in the presence of proteinase K. DNA concentration and purity were checked with NanoDrop One (Thermo Scientific). Quantification of OsHV-1 and total *Vibrio* 16S rDNA was performed using quantitative PCR (qPCR). All amplification reactions were performed using a Roche LightCycler 480 Real-Time thermocycler (qPHD-Montpellier GenomiX platform, Montpellier University, France). A Labcyte Acoustic Automated Liquid Handling Platform (ECHO) was used for pipetting into the 384-well plate (Roche). The total qPCR reaction volume was 1.5 μl with 0.5 μl DNA (40 ng μl^− 1^) and 1 μl LightCycler 480 SYBR Green I Master mix (Roche) containing 0.5 μM PCR primer (Eurogenetec SA). Virus-specific primer pairs targeted a DNA polymerase catalytic subunit (DP, ORF100, AY509253): Fw-ATTGATGATGTGGATAATCTGTG and Rev-GGTAAATACCATTGGTCTTGTTCC [43]. Total *Vibrio* specific primer pairs were Fw-GGCGTAAAGCGCATGCAGGT and Rev-GAAATTCTACCCCCCTCTACAG [44]. qPCR reactions were performed with the following program: 95 °C for 10 min, followed by 40 cycles of denaturation at 95 °C for 10 s, hybridization at 60 °C for 20 s) and elongation at 72 °C for 25 s). After these PCR cycles a melting temperature curve of the amplicon was generated to verify the specificity of the amplification. Absolute quantification of OsHV-1 and total *Vibrio* 16S rDNA copies were calculated by comparing the observed Cq values to a standard curve generated from the DNA polymerase catalytic subunit or from the 16S rDNA of *Vibrio tasmaniensis* LGP32 amplification products cloned into the pCR4-TOPO vector.

## Supplementary information

**Additional file 1. **OsHV-1 and total vibrio colonization in oysters during the two mesocosm experiments. OsHV-1 and total vibrio quantifications in mesocosm experiments from infectious environments of Atlantic or Mediterranean origins at the beginning of the experiments (T0 h) and before the first mortalities (T72 h) in susceptible (S: S_F11_, S_F14_, S_F15_) and resistant (R: R_F21_, R_F23_, R_F48_) oyster families. (**A**) The OsHV-1 load was quantified by qPCR and expressed as viral genomic units per ng of oyster DNA. (**B**) The total 16S vibrio load was quantified by qPCR and expressed as 16S copy number per ng of oyster DNA. Dots represent distinct pools of 10 oysters and bars represent the mean ± SD.

**Additional file 2. **List of DEGs shared by at least two resistant families. This table presents the Log2 fold change of each CGI differentially expressed (significant) in the three resistant oyster families (R_F21_, R_F23_ and R_F48_) compared to the three susceptible families (S_F11_, S_F14_ and S_F15_), the Blast results, the enriched functional categories and details of each comparison (Log2 fold change, *p*-value and number of reads).

**Additional file 3.** List of DEGs specifics to the R_F21_ oyster family. This table presents the Log2 fold change of each CGI differentially expressed (significant) only in the the R_F21_ oyster family compared to the three susceptible families (S_F11_, S_F14_ and S_F15_), the Blast results, the enriched functional categories and details of each comparison (Log2 fold change, p-value, GO categories and number of reads).

**Additional file 4.** List of DEGs specifics to the R_F23_ oyster family. This table presents the Log2 fold change of each CGI differentially expressed (significant) only in the the R_F23_ oyster family compared to the three susceptible families (S_F11_, S_F14_ and S_F15_), the Blast results, the enriched functional categories and details of each comparison (Log2 fold change, p-value, GO categories and number of reads).

**Additional file 5.** List of DEGs specifics to the R_F48_ oyster family. This table presents the Log2 fold change of each CGI differentially expressed (significant) only in the the R_F48_ oyster family compared to the three susceptible families (S_F11_, S_F14_ and S_F15_), the Blast results, the enriched functional categories and details of each comparison (Log2 fold change, p-value, GO categories and number of reads).

**Additional file 6 **Blast2GO annotation of the 28,027 genes identified in the genome of *C. gigas*.

## Data Availability

RNA-seq data have been made available through the SRA database (BioProject accession number PRJNA423079 with submission ID from SRR10799809 to SRR10799844). Complementary information is available from the corresponding authors on reasonable request.

## Abbreviations

2′,5′-OAS: 2′,5′- oligoadenylate synthase
ADAR: dsRNA-specific adenosine deaminase
ADAT: tRNA adenosine deaminases
CGI: *Crassostrea gigas* identifier
DEGs: Differentially expressed genes
HSP: Heat shock proteins
IFI44: Interferon Induced Protein 44
IRAK: Interleukin-1 receptor-associated kinase
IRF: Interferon regulatory factor
JAK: Signal transducers and activators of transcription
MyD88: Myeloid differentiation primary response 88
NFκB: Nuclear Factor Kappa B
PARP: Poly [ADP-ribose] polymerases
POMS: Pacific oyster mortality syndrome
QTL: Quantitative trait loci
RIG-I: Retinoic acid-inducible gene I
RLR: RIG-I-like receptors
SAMHD1: Deoxynucleoside triphosphate triphosphohydrolase
SOCS: Suppressors of cytokine signaling
SR: Scavenger receptors
STAT: Signal transducer and transcription activator
STING: Stimulator of interferon genes
TBK1: Serine/Threonine-Protein Kinase
TLR: Toll-like receptor
TNF: Tumor Necrosis Factor
TRAF: TNF receptor-associated factor
TRIM: Tripartite Motif containing proteins
